# Substance Use is Associated With Frequent Emergency Department Visits in Cardiac Patients

**DOI:** 10.5811/westjem.48499

**Published:** 2025-07-18

**Authors:** Tai Metzger, David A. Berger, Ramin Homayouni

**Affiliations:** * Oakland University William Beaumont School of Medicine, Department of Foundational Medical Studies, Rochester, Michigan; † Corewell Health William Beaumont University Hospital, Department of Emergency Medicine, Royal Oak, Michigan

## Background/Objectives

Social and behavioral determinants of health (SBDoH) influence healthcare utilization and a variety of health outcomes, including among cardiovascular disease (CVD) patients. Currently, the majority of outpatient and emergency department (ED) patients are not adequately screened although their needs may be documented in the clinical notes. The use of artificial intelligence (AI) text processing algorithms to analyze vast amounts of data in the electronic health record (EHR) may provide a more comprehensive view of SBDoH needs across a patient population. AI, which is already used in EDs to provide initial machine read of electrocardiograms, best practice alerts, and detection of hemorrhage on stroke head CT readings, may also facilitate assessment of patient risk of high ED use. Our objective was to apply a novel natural language processing (NLP) approach that we developed to determine which SBDoH are associated with frequent ED use for patients with CVD.

## Methods

We included patients 18–65 years with a history of atrial fibrillation, acute myocardial infarction, ischemic heart disease, or non-ischemic heart disease during a one-year period (9/1/2022–8/31/23) at a large metropolitan hospital in southeast Michigan. Patients over 65 years old were excluded to focus on younger and healthier patients whose visits may be more affected by SBDoH. We used a custom algorithm to combine ICD-10 codes, SDoH screening responses, and SBDoH detected from the clinical notes with NLP. SBDoH factors were compared between high- (≥ 5 visits/year) and non-high utilizers (< 5 visits/year). Logistic regression with backward selection was used to find significant associations between high ED use and demographics, chronic conditions and 17 different SBDoH factors.

## Results

A total of 4,844 patients met inclusion criteria, with 526 (10.9%) having high ED use. Univariate analysis comparing high and low ED use showed significant differences in sex, race, payer mix, average number of chronic conditions, and average number of SBDoH factors (Table 1). Multivariable regression revealed female sex, African American race, financial strain, unreliable transportation, inadequate support system, uninsured/underinsured, medication affordability concerns, depression, alcohol, and opioid abuse were significantly associated with high ED utilization (Table 2). In particular, patients with documented opioid abuse (adjOR 3.25, 95% CI 2.60–4.07, p< .0001) and alcohol abuse (adjOR 2.22, 95% CI 1.75–2.84, p< .0001) had significantly increased odds of frequent ED use. Payer mix was not included in the regression analysis because of the high degree of correlation between Medicaid status and SBDoH factors.

## Conclusions

Using our unique NLP approach, patients with CVD and specific SBDoH factors were associated with high ED use. Consistent with other studies, we found that alcohol and opioid abuse were associated with two- and three-fold higher rates of ED use, respectively. Importantly, substance abuse is not screened in the standard SDoH tools, which emphasizes the importance of aggregating these data from multiple sources within the EHR, including the clinical notes. Future work could consider whether strategically addressing SBDoH, particularly substance use disorder among CVD patients, could reduce high ED use.

## Figures and Tables

**Table 1:** Characteristics of cardiovascular disease patients who frequently (≥5) visited the ED during a one year period at Corewell Health Royal Oak Hospital.

Variable	All	<5 ED visits	≥5 ED visits	p-value*
Sample, n (row %)	4844	4318 (89.1)	526 (10.9)	
Age, median (IQR)	56 (11)	56 (12)	54 (12.5)	< .0001
Female, n (col %)	2093 (43.2)	1807 (41.9)	286 (54.4)	< .0001
Race, n (col %)				
White	2737 (56.6)	2473 (57.4)	264 (50.2)	
Black/AA	1706 (35.3)	1464 (34.0)	242 (46.01)	
Asian	107 (2.2)	99 (2.3)	8 (1.5)	
Other	288 (6.0)	276 (6.4)	12 (2.28)	
Insurance class, n (col %)				< .0001
Commercial	2297 (47.4)	2180 (50.5)	117 (22.2)	
Medicaid	1314 (27.1)	1127 (26.1)	187 (35.6)	
Medicare	1178 (24.3)	957 (22.16)	221 (42.0)	
Other	55 (1.1)	54	1 (0.19)	
Number of SBDH, mean (SD)	1.51 (1.6)	1.36 (1.4)	2.75 (2.1)	< .0001
Chronic Conditions, mean, SD)	5.8 (2.8)	5.59 (2.7)	7.61 (2.9)	< .0001

* p-values were determined using Chi-Square or Fisher’s Exact test for categorical variables and Wilcoxon rank sum test for continuous variables.

*CVD*, cardiovascular disease; *ED*, Emergency Department; *IQR*, interquartile range; *AA*, African American; *SBDH*, social and behavioral determinants of health; *SD*, standard deviation.

**Table 2:** Linear regression analysis of SBDoH associated with high ED use.

Category	Adj OR	95% CI	p-value
Opioid abuse (binary)	3.25	2.60–4.07	< .0001
Alcohol abuse (binary)	2.22	1.75–2.84	< .0001
Depression (binary)	1.88	1.53–2.31	< .0001
Race (Black compared to White)	1.66	1.35–2.04	< .0001
Sex (female compared to male)	1.54	1.26–1.90	< .0001
Financial strain (binary)	1.96	1.42–2.72	< .0001
Uninsured or Under-insured (binary)	2.71	1.58–4.64	< .0003
Unreliable transportation (binary)	2.24	1.39–3.61	< .0009
Inadequate support system (binary)	2.55	1.43–4.55	< .0015
Medication affordability concerns (binary)	2.95	1.39–6.30	< .005

*SBDoH*, social and behavioral determinants of health; *ED*, Emergency Department, *Adj OR*, adjusted odds ratio; *CI*, confidence interval.

